# Systematic Review of Epigenetic Effects of Pharmacological Agents for Bipolar Disorders

**DOI:** 10.3390/brainsci7110154

**Published:** 2017-11-18

**Authors:** Laura E. Lockwood, Nagy A. Youssef

**Affiliations:** 1 Department of Psychiatry & Behavioral Neurobiology, University of Alabama at Birmingham, 1720 7th Avenue South, Birmingham, AL 35233, USA; llockwood@uabmc.edu; 2 Department of Psychiatry & Health Behavior, Medical College of Georgia at Augusta University, 997 St. Sebastian Way, Augusta, GA 30912, USA

**Keywords:** epigenetics, epigenomics, antipsychotics, mood stabilizers, bipolar disorders, mood disorders, drug development

## Abstract

Epigenetic effects of medications are an evolving field of medicine, and can change the landscape of drug development. The aim of this paper is to systematically review the literature of the relationship between common medications used for treatment of bipolar disorders and epigenetic modifications. MedLine/PubMed searches were performed based on pre-specified inclusion criteria from inception to November 2017. Six animal and human studies met the inclusion criteria. These studies examined the epigenetic changes in the main classes of medications that are used in bipolar disorders, namely mood stabilizers and antipsychotics. Although these initial studies have small to moderate sample size, they generally suggest an evolving and accumulating evidence of epigenetic changes that are associated with several of the medications that are used in bipolar I and II disorders. In this manuscript, we describe the specific epigenetic changes that are associated with the medications studied. Of the studies reviewed, five of the six studies revealed epigenetic changes associated with the use of mood stabilizers or antipsychotic medications. This review contributes to future research directions. Further understanding of the complexities of the epigenome and the untangling of the effects and contributions of disease states versus medications is crucial for the future of drug design and the development of new therapeutics. Epigenetic therapeutics hold great promise for complex disease treatment and personalized interventions, including psychiatric diseases.

## 1. Introduction

Epigenetics is an important emerging area of medical research. Epigenetics is “the study of any potentially stable and, ideally, heritable changes in gene expression or cellular phenotype that occurs without changes in Watson-Crick base pairing [1]”. “An epigenetic system should be heritable, self-perpetuating, and reversible” [2]. In other words, epigenetics involves functional changes to the gene without any sequence changes.

Previously, we have systematically reviewed the literature of how epigenetics are involved in depression and suicidality [3]. Our prior paper indicated that disease processes, such as depression, can have epigenetic effects. Abdomalaky et al., point out epigenetic changes in schizophrenia [4]. Zannas et al., have noted epigenetic changes in posttraumatic stress disorder [5]. Epigenetic changes have even been found in bona fide neurodegenerative disorders, such as Alzheimer’s disease, Parkinson’s disease, and Huntington’s disease [6].

There appear to be epigenetic contributions to the genesis and course of bipolar disorder, which are continuing to be further elucidated [7]. It demonstrated that these effects can also be passed transgenerationally. Data indicated that children of parents with bipolar disorder in the United States have a substantially increased risk of being diagnosed with bipolar disorder, and when this is expanded to include all psychiatric diagnoses, 74% of offspring had a diagnosis (as compared to 48% in the general population) [8].

In this systematic review of the literature, we examine if there is an association between epigenetic changes and some of the bone fide medications that are used to treat bipolar disorders. Our hypothesis is that several psychotropic medications that are mainstay for treating bipolar disorders have epigenetic effects. If this proves to be the case, this can have important implications, including the development of psychotropics that engage/correct dysfunctional epigenetic targets in bipolar disorders. This is not surprising, given the already ongoing efforts of developing “epigenetic drugs” for cancer [9], multiple sclerosis [10], and some other diseases. This could also well be the case for potentially disabling bipolar disorders; and it is possible that with knowledge of epigenetic mechanisms, “epigenetic drugs” could be the next forefront of psychopharmacology.

Objective: The objective of this manuscript is to systematically review the literature regarding the association of common medications used for treatment of bipolar disorders with changes in the epigenetic based on pre-specified inclusion criteria.

## 2. Materials and Methods

Inclusion criteria for this review include (1) papers with original data of animal and human studies (2) papers written in English (3) papers that clearly identify the effect/association of one or few separate medications of bipolar disorder on epigenetic changes. Exclusion criteria were review papers and papers written in languages other than English, and non-original data papers.

MedLine/PubMed search was performed from inception to 1 November 2017 using the terms (epigenetics OR epigenomics) AND (mood stabilizers), which yielded 11 results. Of these, seven were excluded due to non-original data papers, and one was excluded due to not being pertinent. Three articles were included in this review. Another search was conducted using the terms (epigenetics OR epigenomics) AND (antipsychotics), which yielded 37 results. Of these, 22 were excluded due to being non-original data papers and 12 were excluded due to not being pertinent. Three were included herein.

Next, more searches were performed for individual drugs to ensure that all of the relevant papers were included in this review. Searches were performed in the same manner as above (for instance, (epigenetics OR epigenomics) AND (amisulpride)). Searches for the following drugs yielded zero results: amisulpride, asenapine, blonanserin, cyamemazine, flupentixol, iloperidone, loxapine, lurasidone, mesoridazine, molindone, oxcarbazepine, paliperidone, perospirone, perphenazine, pipothiazine, sertindole, thiothixene, ziprasidone, zotepine, and zuclopenthixol. Aripiprazole yielded one result, which was excluded because it was a case report. Carbamazepine yielded five results, of which one was a review, three were not pertinent, but one was included in this review. Chlorpromazine yielded two results, of which one was a review and one was not pertinent. Clozapine yielded six results, of which four were reviews, one was not pertinent, and one was included in this review. Fluphenazine yielded one result, which was excluded because it was not pertinent. Haloperidol yielded seven results, of which one was a review, four were not pertinent, and one was included in this review. Lamotrigine yielded one result, which was included. Lithium yielded 18 results, of which nine were reviews, two were in a foreign language, three were not pertinent, and five were included. Olanzapine yielded seven results, of which one was a review, two were not pertinent, and four were included. Quetiapine yielded three results, of which one was a review, one was a case report, and one was included. Risperidone yielded one result, which was excluded because it was a case report. Sulpiride yielded one result, which was not pertinent. Thioridazine yielded two results, which were excluded because one was a case report and the other was not relevant. Topiramate yielded three results, which were not pertinent. Trifluoperazine yielded two results, which were not pertinent. Valproate yielded 105 results, of which 24 were reviews, 78 were not pertinent, one was in a foreign language, and two were included.

**Figure 1:**
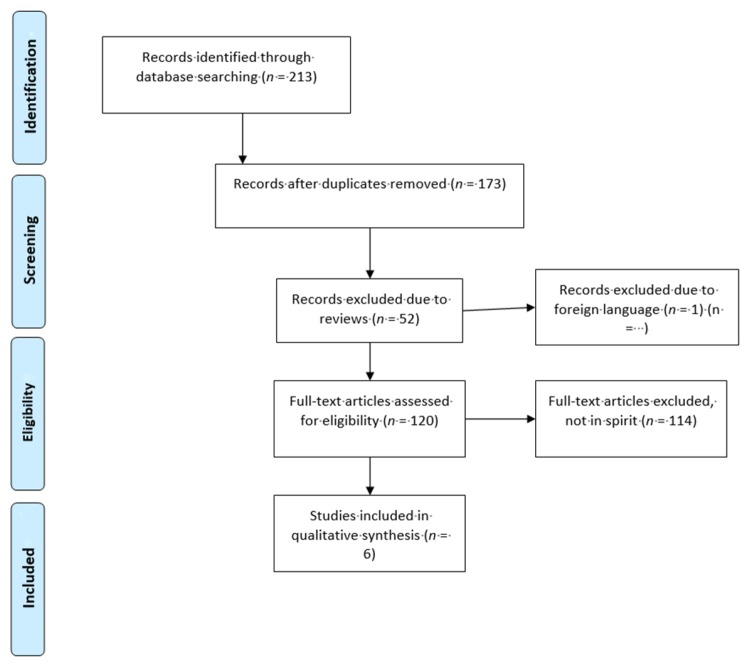
PRISMA flow diagram of studies examining the epigenetic effects of psychopharmacological agents for treating bipolar disorders.

Please refer to the PRISMA diagram (Figure 1) for further details regarding the process of how papers were selected for this review. Studies which were excluded from this systematic review.

## 3. Results

The studies included in the review are described below. They are divided in subsections of mood stabilizers and antipsychotics, being the two main classes of medications that are commonly used to treat bipolar disorder. Table 1 Sumarize the studies examining the epigenetic effects of psychopharmacological agents for treating bipolar disorders.

### 3.1. Mood Stabilizers

Lee et al., investigated epigenetic effects of lithium and valproic acid when administered to rats [11]. The investigators gave the rats food with either lithium, valproic acid, or standard laboratory chow for 30 days, and then collected hippocampal samples at the end of the period. They also collected whole blood samples weekly during the thirty-day period, which were collected from the rats’ tails. At week four, the lithium levels were 1.0 in the lithium group and the valproic acid levels were 37.9 in the valproic acid group. The investigators focused on leptin upregulation (the Lepr gene). Initially, the researchers looked for histone modifications in Lepr expression in a rat hippocampal cell line and found 32.6% increase in lithium treated cells and 127.4% increase in valproic acid treated cells. They were able to find a similar increase in Lepr expression when looking at the hippocampal cells from the rats who had been treated with lithium and valproic acid for 30 days.

The epigenetic alternations in this study may explain the mechanism of action (at least in part) for lithium and valproic acid. However, this is a very preliminary study from a rat model, and it is not clear if it would translate to human subjects. Also, it is not clear if the effect seen is a side effect (perhaps metabolic side effect related to leptin) of the mood stabilizing effect.

Soeiro-de-Souza et al., examined telomerase activity in patients with bipolar I and II disorders who were treated with lithium [12]. The researchers had 28 patients, 39% with bipolar I and 61% with bipolar II, as well as 23 control subjects. The subjects with bipolar disorder were given monotherapy with lithium at an average blood level of 0.48 for six weeks. Peripheral blood mononuclear cells were collected, and from it telomerase activity was evaluated. Telomerase activity did not vary between bipolar I or II disorders or control groups at baseline or at the endpoint. However, there was a negative correlation between the improvement of depressive symptoms after lithium treatment and telomerase activity.

This study appears to be the first to examine telomerase activity in bipolar disorder. As such, it raises interesting questions regarding the mechanism of disease, as well as that of lithium. However, it is also preliminary in nature given the small sample size. The study also did not assess for potential lifestyle factors among participants, such as smoking and diet, which could have confounded the outcome.

Dell’Osso et al., investigated epigenetic effects at Brain-Derived Neurotrophic Factor (BDNF) for bipolar disorder types I and II and major depressive disorder [13]. The researchers investigated methylation during different mood states (manic, hypomanic, mixed, euthymic, and depressed) and looked at the effects of valproic acid and lithium on BDNF modulation. The study involved 43 subjects with major depressive disorder, 61 with bipolar disorder type I, and 50 with bipolar disorder type II, as well as 44 controls. BDNF levels were tested through peripheral blood samples (peripheral blood mononuclear cells). The researchers found that, in a mixed or manic state BDNF methylation levels approximated that of the control subjects. Patients in euthymic or depressed states had significantly higher BDNF methylation levels. Lithium and valproate both decreased BDNF methylation levels, but not to a degree of statistical significance.

This study may also suggest another possible mechanism by which lithium and valproic acid work. The study not only looked at disease states but examined specifically mood states, which was a strength. Though the decrease in BDNF methylation was not shown to a statistically significant level, it is possible that the level could reach significance with a larger sample size.

Houtepen et al., investigated the epigenetic effects of six medications that were used in treating patients with bipolar disorder: lithium, valproic acid, quetiapine, olanzapine, carbamazepine, and lamotrigine [14]. The investigators looked at 169 patients with bipolar I disorder and three with bipolar II disorder who were being treated with monotherapy of the above drugs. Sixty five percent of the patients were on lithium, 19% were on valproic acid, 17% on quetiapine, 16% on olanzapine, 9% on carbamazepine, and 8% on lamotrigine. Peripheral whole blood samples were drawn and methylation levels were tested at the following genes: RELN, SLC1A2, MTNR1A, IGF2, H19, BDNF, SLC6A4, and GAD1. Increased methylation levels were seen in those treated with valproic acid and quetiapine.

This study is interesting in that it compares epigenetic methylation among six drugs that are commonly used for bipolar disorder. It does have some limitations. First, no lithium, valproic acid, or carbamazepine levels were checked, and therefore it is difficult to know roughly how these drugs were dosed, if the levels were therapeutic, or how many participants were adherent to the medications. Second, it is unclear if blood samples were taken both before and after drug administration or only after drug administration in order to measure the changes in methylation status. Finally, while only valproic acid and quetiapine showed a methylation increase in the genes that were tested, it is possible that if other genes had been tested, methylation differences would have been seen with the other drugs.

### 3.2. Antipsychotics

Melka et al., investigated the epigenetic effects of olanzapine in rats. They treated two rats with injections of olanzapine for 19 days and injected two control rats with saline for 19 days [15]. On the 20th day, the rats were euthanized, and DNA samples were collected from the cerebellum, hippocampus, and liver. Of the genes investigated, 19 of 40 showed significant methylation differences in the rats that were treated with olanzapine. These mainly represented increases in methylation in the hippocampus, though a few other tissue-specific changes were found. Many of these genes were found to be related to dopamine expression.

This study is preliminary, as is evident by the small sample size. However, it did find many changes in dopamine expression secondary to olanzapine administration. The mechanism of action of olanzapine has not been fully elucidated yet, but this study suggests that epigenetic changes could be instrumental to the effect seen from olanzapine.

Melka et al., expanded on the above study to further investigate the epigenetic effects of olanzapine in the cerebellum, liver, and hippocampus of rats [16]. The methods were the same as in the Melka et al., [15] study except for that the investigators injected eight rats with olanzapine and eight control rats with saline. As in their previous study, the investigators found that the changes in methylation that were seen were tissue specific. In the hippocampus, there was a significant increase methylation at the dopamine-DARPP32 pathway. There was also a decrease in methylation at CDC42. In the cerebellum, increased methylation was seen in ephrin receptor signaling. Pathways that showed significant methylation differences in the liver were seen to be involved in lipid metabolism, cell death, and organ morphology.

This study further expanded upon the Melka et al. [15] study and confirmed the results that were obtained there. It clarified some of the epigenetic changes that were seen from administering olanzapine. The epigenetic changes found in the liver may account for some of the metabolic side effects resulting from olanzapine administration.

## 4. Discussion

The aim of this study was to determine if the mainstay psychopharmacological agents that treat bipolar disorders are associated with (and possibly work by) epigenetics changes in the genome. Of the six studies reviewed herein, five demonstrated a connection between the two (the 8th study, Dell’Osso et al., 2014 [13], showed a trend toward significance). Many different drugs were investigated in the studies that were reviewed: lithium, valproic acid, lamotrigine, carbamazepine, olanzapine, and quetiapine. These represent drugs from different classes and different mechanism of actions in term of neurotransmitters but all of them represent main treatments of bipolar disorders, including maintenance treatment. We found that many of these “bipolar” medications are associated with epigenetic changes.

Interestingly, the epigenetic effects may not only be associated with the action of the medications, but may also be associated/contribute to some/certain side effects. For instance, Lee et al., 2015 noted an increase in leptin in rats that were treated with valproic acid. This can subsequently increase fat content [17]. Therefore, it is hard to ascertain whether the effect that Lee et al., noted was pointing to the weight gain that is sometimes associated with valproic acid use, or is associated with the mechanism of action of valproic acid, or both. As patients with schizophrenia have a lower life expectancy and higher all-cause mortality than the general population [18], another important area of research (beside mechanistic understating of the action and “epigenetic drug” development) is to further understand these side effects, such as weight gain of antipsychotics at the epigenetic level, in an effort towards ameliorating or preventing these side effects.

Data is emerging that stages of bipolar disorder can potentially be identified in patients and treated in a distinct manner to achieve better patient care [7]. One of the practical implications of discovering epigenetic effects of different medications is to further guide the staging and treatment of patients with bipolar disorder in order to provide the most personalized and best care possible. It appears from research that epigenetic changes occur in the brain based on different stages of the disease. For instance, there appears to be oxidative stress, decreased BDNF levels, and telomere shortening in the later stages of bipolar disorder [19]. By combining knowledge of the epigenetic effects of bipolar illness with knowledge of epigenetic effects of mood stabilizers and antipsychotics, it may become easier to find more patient-centered approaches to pharmacotherapy.

Some of the limitations of this review include that many of the studies that were reviewed herein were preliminary in nature and larger sample sizes are needed to replicate and confirm the findings that are reported. It is possible that publication bias existed, where studies showing lack of statistical significance were not published. Also, the literature contains more animal studies than human studies, and more data needs to be collected in humans to demonstrate applicability to humans.

In conclusion, the current literature shows that several of the reviewed psychiatric medications have epigenetic effects. Further understanding of the complexities of the epigenome and untangling of the effects and contributions of disease states versus interventions is crucial for the future of drug design and the development of new therapeutics. In this paper, we reviewed and summarized the current state of the literature on common psychotropic classes to stimulate further study and provide guidance for future research directions, as well as further appreciation of the epigenomic landscape. Epigenomic therapeutics hold great promise for complex disease treatment and personalized interventions, including psychiatric diseases.

## Figures and Tables

**Table 1:** Studies examining the epigenetic effects of psychopharmacological agents for treating bipolar disorders.

Studies	Number of Subjects	Sample Studied	Medication(s)/Gene(s) Targeted or Brain Region(s) with Epigenetic Changes	Epigenetic Difference Found?	Animal or Human	Comments
Lee et al., 2015 [11]	12 Lithium, 12 Valproic acid, 12 control	Whole blood from rats’ tails	Lithium & Valproic Acid/Lepr	Yes (Lithium *p* = 0.013, valproic acid *p* = 0.008)	Animal	Lepr gene;
Soeiro-de-Souza et al., 2014 [12]	28 subjects, 23 controls	Peripheral blood mononuclear cells	Lithium/Telomerase activity	Yes (*p* = 0.007)	Human	Telomerase activity and Lithium
Dell’Osso et al., 2014 [13]	44 subjects with major depressive disorder, 61 with bipolar I disorder, 50 with bipolar II disorder, and 44 controls; 16 patients on Lithium, 17 patients on Valproic acid	Peripheral blood mononuclear cells	Lithium and valproic acid/BDNF	Not statistically significant for Lithium & valproic acid (no *p*-value reported)	Human	BDNF methylation significantly higher in bipolar I than MDD *p* < 0.01; BDNF methylation significantly higher in mixed/manic than depressed state *p* < 0.01
Houtepen et al., 2016 [14]	172:112 patients on Lithium, 33 patients on Valproic acid, 29 patients on Quetiapine, 28 patients on Olanzapine, 15 patients on Carbamazepine, and 14 patients on Lamotrigine (some patients on more than one medication)	Peripheral whole blood samples	Lithium, valproic acid, quetiapine, olanzapine, carbamazepine, & lamotrigine/RELN, SLC1A2, MTNR1A, IGF2, H19, BDNF, SLC6A4, GAD1	Yes for valproic acid & quetiapine only (Quetiapine *p* = 0.040, Valproic acid *p* = 0.0005)	Human	Genes: RELN, SLC1A2, MTNR1A, IGF2, H19, BDNF, SLC6A4, GAD1
Melka et al., 2013 [15]	2 olanzapine, 2 control	DNA samples from the cerebellum, hippocampus, & liver of rats	Olanzapine/methylation increases in the hippocampus	Yes (*p* < 0.01)	Animal	40 dopaminergic genes, including DRD5, SLC18A2, & DDC8
Melka et al., 2014 [16]	8 olanzapine, 8 control	DNA samples from the cerebellum, hippocampus, & liver of rats	Olanzapine/methylation increases in the hippocampus	Yes, *p* = 1.65 × 10^−3^ for Dopamine-DARPP32 feedback in cAMP signaling *p* = 2.52 × 10^−3^ for CDC42 signaling	Animal	Dopamine-DARPP32 feedback in cAMP signaling CDC42 signaling

## References

[1] Goldberg A.D., Allis C.D., Bernstein E. (2007). Epigenetics: A landscape takes shape. Cell.

[2] Riddihough G., Zahn L.M. (2010). Epigenetics. What is epigenetics? Introduction. Science.

[3] Lockwood L.E., Su S., Youssef N.A. (2015). The role of epigenetics in depression and suicide: A platform for gene-environment interactions. Psychiatry Res..

[4] Abdolmaleky H.M., Zhou J.R., Thiagalingam S. (2015). An update on the epigenetics of psychotic diseases and autism. Epigenomics.

[5] Zannas A.S., Provencal N., Binder E.B. (2015). Epigenetics of Posttraumatic Stress Disorder: Current Evidence, Challenges, and Future Directions. Biol. Psychiatry.

[6] Lardenoije R., Iatrou A., Kenis G., Kompotis K., Steinbusch H.W., Mastroeni D., Coleman P., Lemere C.A., Hof P.R., van den Hove D.L. (2015). The epigenetics of aging and neurodegeneration. Prog. Neurobiol..

[7] Berk M., Post R., Ratheesh A., Gliddon E., Singh A., Vieta E., Carvalho A.F., Ashton M.M., Berk L., Cotton S.M. (2017). Staging in bipolar disorder: From theoretical framework to clinical utility. World Psychiatry.

[8] Axelson D., Goldstein B., Goldstein T., Monk K., Yu H., Hickey M.B., Sakolsky D., Diler R., Hafeman D., Merranko J. (2015). Diagnostic Precursors to Bipolar Disorder in Offspring of Parents with Bipolar Disorder: A Longitudinal Study. Am. J. Psychiatry.

[9] Jones P.A., Issa J.P., Baylin S. (2016). Targeting the cancer epigenome for therapy. Nat. Rev. Genet..

[10] Peedicayil J. (2016). Epigenetic Drugs for Multiple Sclerosis. Curr. Neuropharmacol..

[11] Lee R.S., Pirooznia M., Guintivano J., Ly M., Ewald E.R., Tamashiro K.L., Gould T.D., Moran T.H., Potash J.B. (2015). Search for common targets of lithium and valproic acid identifies novel epigenetic effects of lithium on the rat leptin receptor gene. Transl. Psychiatry.

[12] Soeiro-de-Souza M.G., Teixeira A.L., Mateo E.C., Zanetti M.V., Rodrigues F.G., de Paula V.J., Bezerra J.F., Moreno R.A., Gattaz W.F., Machado-Vieira R. (2014). Leukocyte telomerase activity and antidepressant efficacy in bipolar disorder. Eur. Neuropsychopharmacol..

[13] Dell’Osso B., D’Addario C., Carlotta Palazzo M., Benatti B., Camuri G., Galimberti D., Fenoglio C., Scarpini E., Di Francesco A., Maccarrone M. (2014). Epigenetic modulation of BDNF gene: Differences in DNA methylation between unipolar and bipolar patients. J. Affect. Disord..

[14] Houtepen L.C., van Bergen A.H., Vinkers C.H., Boks M.P. (2016). DNA methylation signatures of mood stabilizers and antipsychotics in bipolar disorder. Epigenomics.

[15] Melka M.G., Castellani C.A., Laufer B.I., Rajakumar R.N., O’Reilly R., Singh S.M. (2013). Olanzapine induced DNA methylation changes support the dopamine hypothesis of psychosis. J. Mol. Psychiatry.

[16] Melka M.G., Laufer B.I., McDonald P., Castellani C.A., Rajakumar N., O’Reilly R., Singh S.M. (2014). The effects of olanzapine on genome-wide DNA methylation in the hippocampus and cerebellum. Clin. Epigenetics.

[17] Mantzoros C.S. (1999). The role of leptin in human obesity and disease: A review of current evidence. Ann. Intern. Med..

[18] Azad M.C., Shoesmith W.D., Al Mamun M., Abdullah A.F., Naing D.K., Phanindranath M., Turin T.C. (2016). Cardiovascular diseases among patients with schizophrenia. Asian J. Psychiatry.

[19] Vieta E., Reinares M., Rosa A.R. (2011). Staging bipolar disorder. Neurotox. Res..

